# Arginine-linked HPV-associated E7 displaying bacteria-derived outer membrane vesicles as a potent antigen-specific cancer vaccine

**DOI:** 10.1186/s12967-024-05195-7

**Published:** 2024-04-22

**Authors:** Suyang Wang, Chao-Cheng Chen, Ming-Hung Hu, Michelle Cheng, Hsin-Fang Tu, Ya-Chea Tsai, Jr-Ming Yang, T. C. Wu, Chuan-Hsiang Huang, Chien-Fu Hung

**Affiliations:** 1 Department of Pathology, Johns Hopkins University School of Medicine, 1550 Orleans Street, CRB II 307, Baltimore, MD 21287 USA; 2 Department of Oncology, Johns Hopkins University School of Medicine, 1550 Orleans Street, CRB II 307, Baltimore, MD 21287 USA; 3 Department of Obstetrics and Gynecology, Johns Hopkins University School of Medicine, Baltimore, MD USA; 4 Molecular Microbiology and Immunology, Johns Hopkins University School of Medicine, Baltimore, MD USA

**Keywords:** Bacteria outer membrane vesicle, Antigen display, Cancer vaccine, Tumor antigen-specific T cell, Tumor infiltrating lymphocytes

## Abstract

**Background:**

Bacteria-based cancer therapy have demonstrated innovative strategies to combat tumors. Recent studies have focused on gram-negative bacterial outer membrane vesicles (OMVs) as a novel cancer immunotherapy strategy due to its intrinsic properties as a versatile carrier.

**Method:**

Here, we developed an Human Papillomavirus (HPV)-associated E7 antigen displaying *Salmonella*-derived OMV vaccine, utilizing a Poly(L-arginine) cell penetrating peptide (CPP) to enhance HPV16 E7 (aa49-67) H-2 Db and OMV affinity, termed SOMV-9RE7.

**Results:**

Due to OMV’s intrinsic immunogenic properties, SOMV-9RE7 effectively activates adaptive immunity through antigen-presenting cell uptake and antigen cross-presentation. Vaccination of engineered OMVs shows immediate tumor suppression and recruitment of infiltrating tumor-reactive immune cells.

**Conclusion:**

The simplicity of the arginine coating strategy boasts the versatility of immuno-stimulating OMVs that can be broadly implemented to personalized bacterial immunotherapeutic applications.

**Supplementary Information:**

The online version contains supplementary material available at 10.1186/s12967-024-05195-7.

## Background

Current advanced cancer immunotherapy treatments boost humoral and cellular immunity without the non-specific targets and toxic effects on normal cells as conventional cancer treatment [1, 2]. However, despite these advances, the inability to predict treatment efficacy, the need for additional biomarkers, the development of resistance to cancer immunotherapies, and the high treatment costs continue to serve as a limitation in immunotherapeutic treatments [3, 4]. Therefore, therapy with live tumor-targeting bacteria has received attention as a unique option to overcome these challenges [5]. Studies have shown that bacteria-based therapies can serve as a monotherapy or complement to other anticancer therapies [5–7]. Previously, we showed that live *Salmonella* and cytokine combination therapy induced potent T-cell immunity and long-term tumor control in mice [8]. We also demonstrated that heat-inactivated *Salmonella* (S. typhimurium) could display tumor antigens to achieve tumor-specific immune responses [9]. Nevertheless, a significant limitation of live bacteria lies in its off-target toxicity and lowered efficacy in inactivated bacteria [10]. Therefore, further investigation is needed to develop an approach with bacteria-based therapies that is more potent than heat-inactivated bacteria therapies, while also addressing the safety concerns of using live attenuated bacteria.

In recent years, studies have shown that gram-negative bacteria can naturally release outer membrane vesicles (OMVs), which comprises of lipopolysaccharides (LPS), outer membrane proteins, periplasmic proteins, and phospholipids and can serve as carriers for various substances such as toxins, metabolites, enzymes, virulence factors, and genetic material (DNA and RNA) [11–13]. Unlike attenuated bacteria, OMVs are considered safer and can effectively stimulate the immune system by delivering key immunogens from their parent bacteria [13–15]. Genetic engineering techniques have shown that the construction of recombinant OMVs improves target precision through surface protein and carries exogenous proteins for improved immunogenicity [16, 17]. As a neoantigen vaccine, OMVs can fuse multiple surface proteins and therefore simultaneously display various distinct tumor antigens to elicit a synergistic antitumor immune response in metastatic lung melanoma and subcutaneous colorectal cancer models [18]. These studies have further underscored the impact of bacteria OMVs as a versatile immunotherapeutic approach in developing cancer vaccines, as it presents a balance between immunogenicity and safety [15, 19–21].

Previously, we optimized a bacteria antigen-display strategy through modification of the Human Papillomavirus (HPV)-associated E7 antigen, incorporating nine arginine residues (9RE7) for enhanced E7 coating [8]. Poly-l-arginine is a cell penetrating peptide (CPP), often used for mammalian cell uptake and delivery of drugs or macromolecules such as proteins and enzymes [22, 23]. Here, we coated 9RE7 on *Salmonella* OMV (SOMV), a naturally released OMV derived from *Salmonella* SL7207, and synthesized SOMV-9RE7 which will be investigated to serve as a safer and more effective method to delivery HPV E7 antigen. By generating systemic E7-specific CD8+ T cells and recruiting them to the tumor microenvironment (TME), SOMV-9RE7 exhibited promising antitumor effects. These results demonstrate a broad application of 9RE7 peptide and an alternative to traditional bacteria immunotherapy.

## Material and methods

### Cell preparation

E7-expressing TC-1 tumor cells and dendritic cells were grown in vitro in RPMI 1640 media containing 10% (v/v) fetal bovine serum, 50 units/mL of penicillin/streptomycin, 2 mM L-glutamine, 1 mM sodium pyruvate, 2 mM non-essential amino acids, and 0.1% (v/v) 2-mercaptoethanol under 37 °C with 5% CO2. E7-specific CD8+ splenocytes were isolated from mice vaccinated with E7 DNA and incubated with E7 expressing cells and maintained in culture.

### Bacteria-derived outer membrane vesicle

*Salmonella* SL7207 was grown overnight in LB broth at 37 °C. On the next day, 1 mL of the overnight *Salmonella* culture was added to 9 mL of fresh LB medium and incubated until O.D. 600 reading of 0.5. This freshly cultured *Salmonella* was added to fresh LB medium at 1:100 dilution in 250 mL culture at 37 °C for 16 h. The supernatant was collected after centrifugation and filtered through a 0.45 µm MCE Membrane Filter (Millipore Sigma). Filtered medium was transferred to ultracentrifuge tubes and at 150,000 RPM for 3 h (Beckman). The supernatant was removed and particles at the bottom of the tubes were collected and suspended in 1 mL PBS and stored at − 80 °C. OMV yield is calculated using Bio-Rad protein assay with a concentration of approximately 1.5 mg/mL.

### Peptide synthesis

Peptides used in this study include RRRRRRRRR-RAHYNIVTF (E7 protein amino acids 49–57), termed 9RE7, and was synthesized by GenScript (Piscataway, NJ, USA) at a purity of over 90%.

### SOMV-9RE7 generation and characterization

SOMV-9RE7 is synthesized by combining SOMV and 9RE7 in PBS buffer to be vortexed for 30 min. Subsequent dialysis is performed using a 50kD Amicron Ultra Centrifugal Filter (Millipore Sigma) to remove unbound peptides. For characterization of SOMV-9RE7, 10 µg of SOMV was mixed with 1 µg of FITC conjugated peptides E7 or 9RE7 in the PBS buffer. Bacteria/peptide mixture was vortexed at room temperature for 30 min, followed by dialysis with the 50kD Amicron Ultra Centrifugal Filter (Millipore Sigma) to remove unbound peptides. FITC signals were measured by 13-color B-Y-R-V CytoFLEX S (Beckman Coulter). Particle size and charge was determined by Malvern Zetasizer (Worcestershire, UK). 40% of 9RE7 remained coated on SOMV after dialysis. This was determined by interpolating the standard curve of FITC-labeled SOMV-9RE7 at 500 nm with Nanodrop One (Thermo Fisher Scientific).

### In vitro T cell activation

10 µg of SOMV and 1 µg 9RE7 are used to synthesize SOMV-9RE7 as described above. E7-specific CD8+ T cell activation follows previously established protocol [24, 25]. SOMV-9RE7 is incubated with 1 × 10^5^ dendritic cell line in 96 well plate cultured with complete RPMI media at 37 °C, 5% CO2 overnight. After aspirating culture medium and washing with PBS, 5 × 10^5^ E7-specific CD8+ T cells were added to the dendritic cell line and blocked with Brefeldin A + Monensin Golgi Plug (Thermo Fisher Scientific) overnight. Cells were collected and stained with APC-A750-conjugated anti-mouse CD8α antibody (Biolegend) before permeabilization with eBioscience Fixation (Invitrogen) and intracellular staining FITC-conjugated IFNγ antibodies.

### Mice vaccination

For tumor inoculation, 1 × 10^5^ TC-1 cells in 50 µL of PBS were subcutaneously injected into 6–8 weeks old female C57BL/6 mice at the lower right abdomen. Largest length and width were measured by digital calipers twice per week. Tumor volumes were calculated by the formula: V = (Length × Width^2^)/2. At the indicated time points, TC-1 tumor-bearing mice were vaccinated subcutaneously in the tumor graft region with 10 µg of 9RE7 peptides, 10 µg SOMV, or SOMV-9RE7 (10 µg of SOMV and 10 µg of 9RE7).

### Flow cytometry analyses

Blood samples were collected from vaccinated mice after final treatment. Red blood cell (RBC) lysis using RBC lysis buffer (eBioscience) collected peripheral blood mononuclear cells (PBMC). For tumor tissue sample preparation, tissue was collected from mice and transferred to FACS buffer in gentleMACS C tubes (Miltenyi Biotec). Tissue digestion enzymes including Collagenase I, Collagenase IV, and DNase I were added to samples. Samples were dissociated with gentleMACS Dissociator (Miltenyi Biotec) before incubating for 20 min. After centrifugation and buffer exchange, tumor samples were purified by loading onto Ficoll-Paque Plus (GE Healthcare Life Sciences, Marlborough, MA). Tubes were centrifuged for 20 min and Ficoll-RPMI interface was collected. Samples were then counted, plated at equal cell numbers, and prepared for flow cytometry. Spleen grinded through Corning® 70 μm Cell Strainer (Millipore Sigma) with syringe stopper and suspended in RPMI medium. Next, RBC lysis was performed and splenocytes were counted and plated at appropriate cell numbers.

For FACS analysis, live cells were identified with Zombie Aqua live/dead (BioLegend) and Fc Block to reduce nonspecific antibody binding. Peripheral antigen-reactive CD8+ T cell population in PBMC was identified with PE-conjugated HPV16 E7aa49–57 peptide loaded H-2 Db E7 tetramer and APC-A750-conjugated anti-mouse CD8α antibodies. For tumor infiltrating lymphocyte and splenocyte analyses, we used APC-A700-conjugated anti-mouse CD45 antibodies, BV421-conjugated anti-mouse CD3 antibodies, PE-Cy5-conjugated anti-mouse CD8 antibodies, PE-conjugated HPV16 E7 tetramer, and BV-650-conjugated anti-mouse IFNγ antibodies. FACS analysis was performed using CytoFLEX S (Beckman Coulter Life Sciences) and fluorescent compensation was generated using single-antibody controls. All flow cytometry data and gating strategies were performed by FlowJo software.

### Statistical analysis

GraphPad Prism V.10 software was used to perform data statistical analysis. Data is represented as means and standard error of the mean. Kaplan–Meier survival plots were used to estimate the survival percentage and tumor-free rate. Long rank tests were used to compare the survival time between treatment groups. Comparison between individual data points were analyzed for variance with one-way ANOVA and the Tukey–Kramer multiple comparison test, *p ≤ 0.05, **p ≤ 0.01, *** p ≤ 0.001, **** p ≤ 0.0001, ns = not significant.

## Results

### Poly-arginine anchored E7 peptide can be coated on *Salmonella*-derived outer membrane vesicles as an immunogenic antigen carrier

**Fig. 1:**
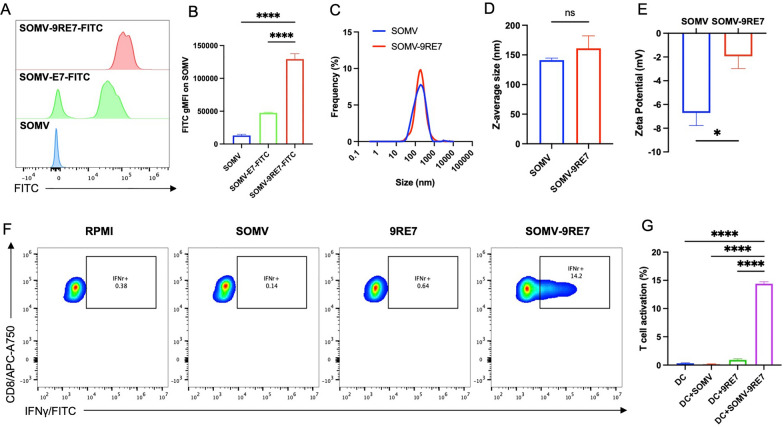
Characterization of 9RE7 peptide presenting *Salmonella-*derived outer membrane vesicle. Nanoscale flow cytometry analysis of binding efficiency between *Salmonella*-derived outer membrane vesicle (SOMV) and nona-arginine extended HPV E7 peptide (9RE7). 10 µg of SOMV is incubated with 1 µg FITC-conjugated E7 peptide (E7-FITC), termed SOMV-E7-FITC, or FITC-conjugated 9RE7 (9RE7-FITC) peptide, termed SOMV-9RE7-FITC, with **A** representative flow cytometry histogram of FITC channel and **B** bar graph of gMFI on peptide coated SOMV. **C** Dynamic light scattering particle size frequency of SOMV and 9RE7-coated SOMV (SOMV-9RE7). **D** Z-average size comparison of SOMV and SOMV-9RE7. **E** Zeta potential measurements of the surface electric charge on SOMV and SOMV-9RE7. **F** Flow cytometry analysis of E7-specific T cell activation by SOMV-9RE7 in vitro. SOMV, 9RE7, and SOMV-9RE7 are prepared and dialyzed with 50kD MWCO Amicon centrifugal filter before incubated with dendritic cell line overnight. Upon replacing culture medium, E7-specific T cells are added to dendritic cells for another 24 h and blocked golgi protein transport inhibitor. After collecting the cell mixture, E7-specific T cells are stained with APC-A750-conjugated anti-mouse CD8α antibody stainings before cell membrane permeation and FITC anti-mouse IFNγ antibody for flow cytometry analyses. Representative flow cytometric images show CD8α and IFNγ gating. **G** Bar graph summary of T cells with positive IFNγ population. *p < 0.05, ****p < 0.0001

First, we wanted to demonstrate that SOMV can bind to 9RE7 peptide efficiently, so we characterized the binding affinity of 9RE7 to SOMV using FITC-conjugated peptides. We were able to visualize the 9RE7-FITC and E7-FITC distribution on SOMV with nanoscale flow cytometry. In the flow cytometry histogram, SOMV coated with 9RE7-FITC had a more homogeneous population than coating with E7-FITC (Fig. 1A). By calculating the mean fluorescence intensity, SOMV-9RE7-FITC had a significantly higher FITC signal than SOMV-E7-FITC, increasing gMFI by three folds and showing the critical role of the 9R moiety in achieving high affinity peptide coating (Fig. 1B). Next, we measured the particle size distributions and surface charges of SOMV and SOMV-9RE7 to elucidate on particle properties. Using dynamic light analysis, we observed a similar size distribution of SOMV as SOMV-9RE7, average zeta size of 132.6 nm and 153.2 nm respectively, with no significant changes in particle size after modification with 9RE7 (Fig. 1C, D). However, there was a significant alteration to the vesicle surface charge, where the negative charge of SOMV had a positive shift by attaching 9RE7 that increased its Zeta-potential from -6.7 mV to -1.9 mV (Fig. 1E). Therefore, we have demonstrated the successful creation of a stable particle, SOMV-9RE7, using the arginine-anchored E7 peptide platform.

Next, we sought to show the immunogenic properties of SOMV-9RE7 as an antigen delivery particle. We prepared SOMV, 9RE7, and SOMV-9RE7 as described in methods prior to co-culturing with dendritic cells in RPMI medium overnight. Then, E7-specific CD8+ splenocytes were added to each group for stimulation overnight. From flow cytometry analysis gating for CD8 and intracellular IFNγ, SOMV-9RE7 treated group showed the highest splenocyte activation, and SOMV alone could not induce IFNγ expression (Fig. 1F, G). Importantly, 9RE7 after centrifugal filtration had no IFNγ expression, meaning that the dialysis protocol effectively removed free peptides. Our results indicated that SOMV-9RE7 can activate antigen presenting cells (APCs) and cross-present Major Histocompatibility Complex (MHC) class I restricted antigen to engage E7-specific CD8+ T cells in vitro.

### Local treatment of SOMV-9RE7 show significant anti-tumor efficacy by inducing adaptive antigen-specific immunity

**Fig. 2:**
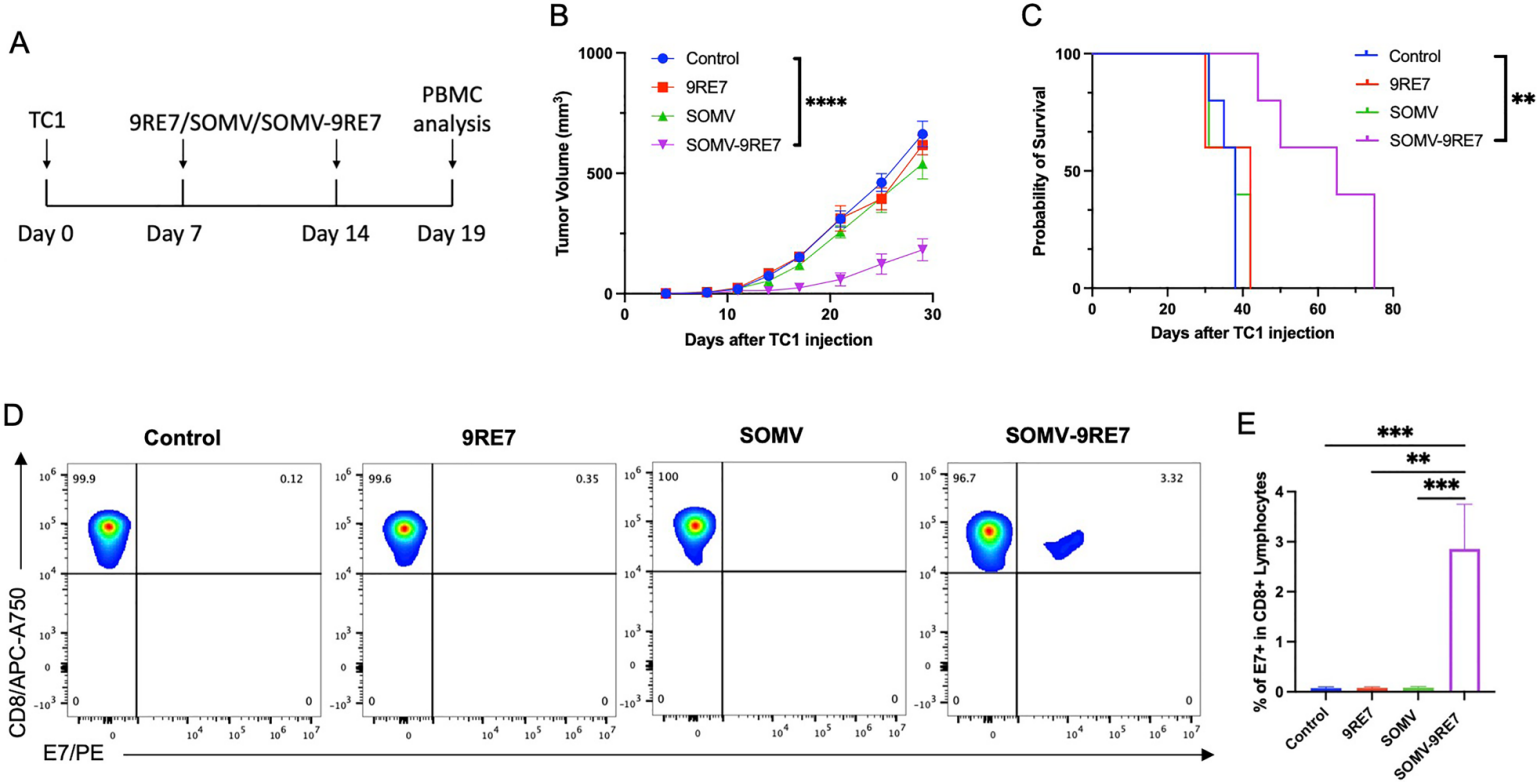
Antitumor efficacy and antigen-specific immune response under SOMV-9RE7 treatment in TC1 tumor-bearing mouse model. **A** Schematic illustration of experiment schedule. C57BL/6 mice are inoculated with 1 × 10^5^ TC-1 cells subcutaneously (s.c.) on day 0. They are given weekly treatments s.c. of either 10 µg 9RE7, 10 µg SOMV, or SOMV-9RE7 (10 µg SOMV incubated with 10 µg of 9RE7) for 2 weeks. PBMC is collected from the mice 5 days after the final treatment to measure. **B** Tumor growth curve of TC-1 tumor-bearing mice. **C** Kaplan–Meier survival curve of TC-1 tumor-bearing mice. **D** Quantification of E7-specific T cell population. PBMC was collected from each group and processed by staining with PE-conjugated HPV16 E7aa49-57 peptide-loaded H-2Db E7 tetramer and APC-A750-conjugated anti-mouse CD8α antibodies. Representative flow images show gating strategy for identifying CD8+ and PE+ T cells for all 4 groups and **E** bar graph representation are shown. **p < 0.01, ***p < 0.001, ****p < 0.0001

We investigated the response of the HPV positive TC-1 model to SOMV-9RE7 treatment. At 7 and 14 days after TC-1 inoculation, we delivered SOMV-9RE7 subcutaneously on the tumor side, while comparing it to 9RE7 and SOMV treatments, as shown in the schema in Fig. 2A. Initial vaccination of SOMV and SOMV-9RE7 caused mild infection responses in the form of weight loss after 1 day, but the vaccinated mice quickly recovered within 3 days after treatment (Additional file 1: Fig. S1). SOMV-9RE7 therapy had a visible impact on tumor growth after the first dose by significantly reducing tumor size compared to the control group, whereas 9RE7 or SOMV treatments had no long-term impact on tumor growth (Fig. 2B). Accordingly, SOMV-9RE7 treated mice had significantly longer survival than the control group by doubling the last survival day (Fig. 2C). PBMC was collected from each group after the second dose to test for development of antigen-reactive lymphocytes. In parallel to the antitumor results, flow cytometry analysis showed a significantly higher population of E7+ cytotoxic T lymphocytes (CTLs) under SOMV-9RE7 treatment (Fig. 2D, E). Tumor antigen 9RE7 was insufficient to induce E7-specific immunity without presence of an adjuvant. These results have shown that SOMV-9RE7 can suppress TC-1 tumor growth through mediating potent E7-specific CD8+ T cell mediated response in vivo.

### SOMV-9RE7 induces effector T cell proliferation and activity in the tumor microenvironment and spleen

**Fig. 3:**
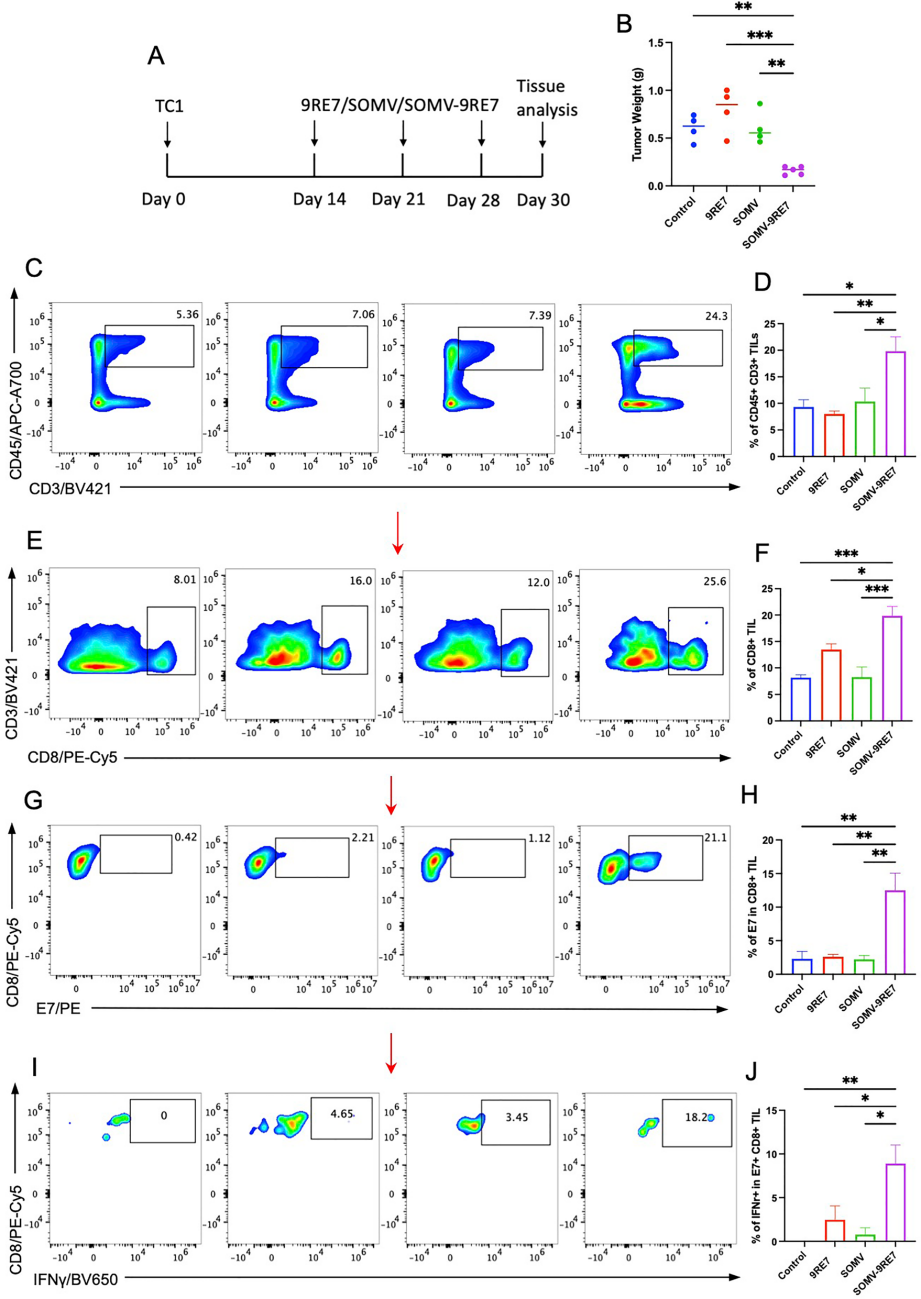
Analysis of tumor infiltrating lymphocytes in SOMV-9RE7 treated TC-1 tumor-bearing mouse model. **A** Treatment schedule of C57BL/6 mice inoculated with 1 × 10^5^ TC-1 cells s.c. and treatments are given on day 14 for three doses. Tumor and spleen from each group are harvested on day 30. **B** Tumor weight of each treatment group after harvestation. Treated tumors are processed and isolated for mononuclear cells. Total TIL populations are identified by staining for APC-A700-conjugated anti-mouse CD45 and BV421-conjugated anti-mouse CD3 antibodies. Representative flow cytometry images show **C** CD45+ and CD3+ positive lymphocyte population and **D** bar graph analysis of CD45+ and CD3+ TIL percentages of each treatment. **E** CD8+ T cell subpopulation in CD3 + /CD45 + cells are identified with PE-Cy5-conjugated anti-mouse CD8 antibody and **F** bar graph analysis of CD8+ TIL population. Then, PE-conjugated HPV16 E7 tetramer are used to quantify E7-specific TIL with **G** gating strategy for E7 positive cytotoxic T cells and **H** bar graph analysis of E7 percentage. **G** E7-specific effector T cell functionality is determined by intracellular staining with BV650-conjugated IFNγ antibody, and **J** calculation of IFNγ expression in each group

To analyze the effect on SOMV-9RE7 on the organ systems, we adjusted the treatment schedule. 14 days after TC-1 inoculation, we initiated treatment for three doses before harvesting tumor and spleens from each group (Fig. 3A). The SOMV-9RE7 treated mice had significantly smaller tumors in weight compared to the other groups (Fig. 3B). Tumors were processed into single mononuclear cells for FACS analysis where we saw the highest CD45+ CD3+ lymphocyte accumulation in SOMV-9RE7 treated TME, whereas SOMV treatment did not induce lymphocyte trafficking in the TME (Fig. 3C, D). Within the total tumor infiltrating-lymphocytes (TILs), we examined the CD8+ CTL population (Fig. 3E). The 9RE7 peptide treatment had a higher CD8+ population than the control or SOMV group, but peptide-coated vesicle SOMV-9RE7 had significantly more CTLs than the other groups (Fig. 3F). More importantly, upon examining antigen-specific CTLs staining with HPV16 E7 tetramer (Fig. 3G), there was only a small E7 positive populations in control, 9RE7, and SOMV groups compared to SOMV-9RE7, which had a significant E7-specific CTL population (Fig. 3H). Finally, we examined E7-specific effector functional activity and saw significantly more IFNγ expression in SOMV-9RE7 treated E7+ CD8+ TILs (Fig. 3I, J).

**Fig. 4:**
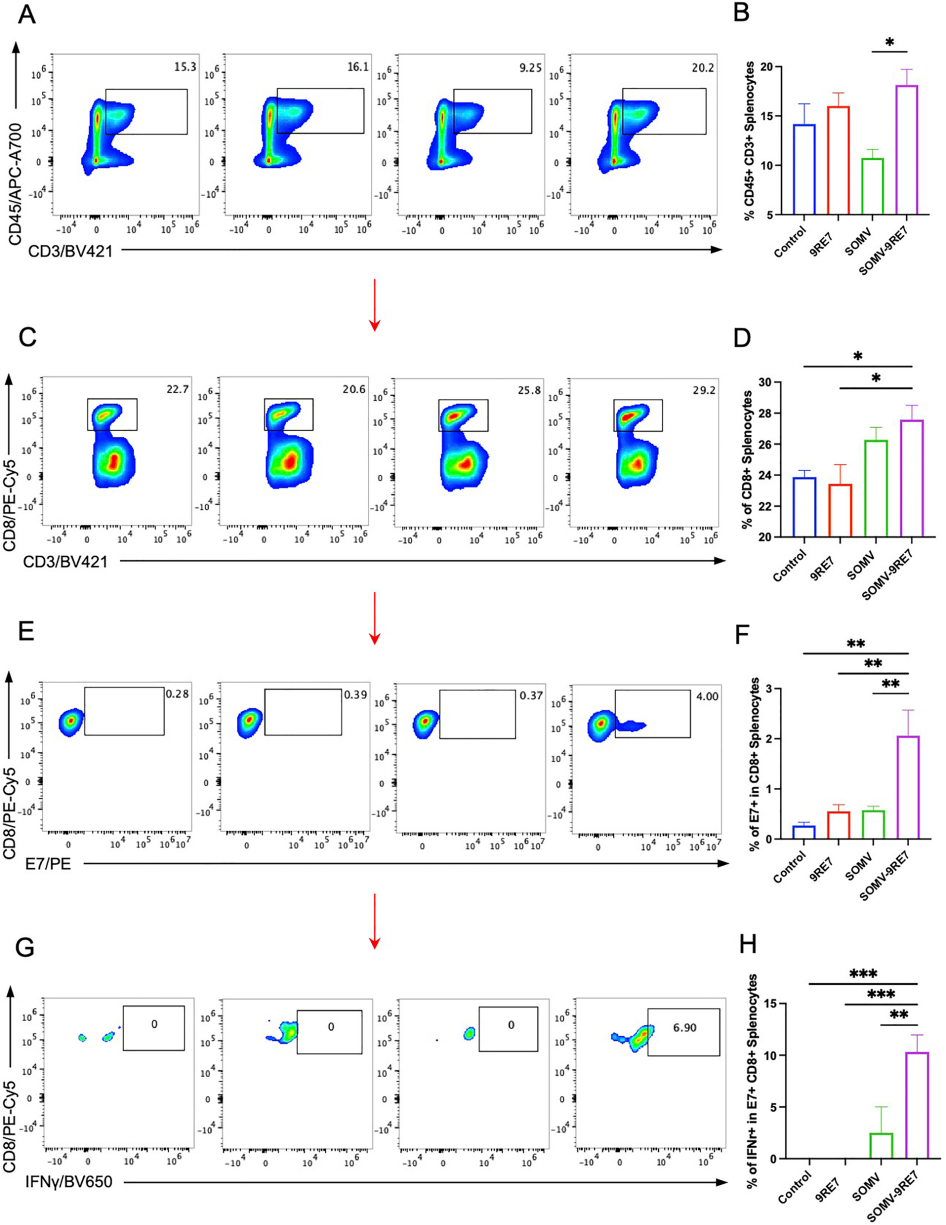
Characterization of splenocyte populations in SOMV-9RE7 vaccinated TC-1 tumor model. **A** Flow cytometry analysis of splenic lymphocyte population from previously described treatments by staining with APC-A700-conjugated anti-mouse CD45 and BV421-conjugated anti-mouse CD3 antibodies. **B** Percentage of CD45+ and CD3+ splenocyte population in each treatment group. **C** Cytotoxic lymphocyte population within splenic lymphocytes is identified by staining with PE-Cy5-conjugated anti-mouse CD8 antibody and **D** bar graph shows CD8+ T cells from each group. **E** Within CD8+ T cells, E7 antigen-specific T cells are gated with PE-conjugated HPV16 E7 tetramer and **F** E7+ populations are analyzed from each group. (**G**) E7-specific CTL effector function is presented by intracellular staining with BV650-conjugated IFNγ antibody and **H** percentage of IFNγ expression with bar graph

At the same time, we analyzed splenocyte populations to compare TIL profile of the TME. Flow cytometry analysis of splenocytes shows a slight increase in CD45+ CD3+ splenic lymphocytes in the SOMV-9RE7 group compared to control mice (Fig. 4A, B). Interestingly, SOMV treated mice spleens showed a significant decrease in total CD45+ CD3+ lymphocytes than SOMV-9RE7 treatment (Fig. 4B). Both SOMV and SOMV-9RE7 had increased CD8+ T cell populations (Fig. 4C, D), with only SOMV-9RE7 treated mice developing a significant E7-specific splenocyte population (Fig. 4E, F). Upon analyzing effector cell activation, SOMV-9RE7 treated E7-specific splenocytes had significantly more IFNγ activity than other groups (Fig. 4G, H). Combining both TIL and splenocyte analysis, SOMV-9RE7 has shown it effectively develops adaptive immunity in the lymphoid system, while actively recruiting E7 antigen-reactive T cells to the TME for tumor-targeting immunity.

## Discussion

In this study, we wanted to demonstrate the wide applicability of our efficient arginine peptide-enhanced antigen coating platform that is not only restricted to bacteria as carriers [8], but also bacteria-derived OMVs. Here, we saw the importance of arginine residues in enhancing affinity between 9RE7 peptide and SOMV. In contrast, FITC-labeled E7 coating of SOMV resulted in heterogeneous populations of SOMV-E7-FITC, indicating non-uniform distribution of E7-FITC on SOMV (Fig. 1A). Further investigation of synthesized SOMV-9RE7 confirmed its 9RE7 presentation due to a positive surface charge increase from SOMV (Fig. 1E). These results suggest that the 9RE7 peptide coating strategy can also be applied to OMVs, demonstrating its diverse utility as an immunotherapeutic method.

By retaining the similar inflammatory components to parental gram-negative bacteria, OMVs can engage APCs through TLR4 recognition and cross-present antigens to T cells [26]. Previously we saw that 9RE7 peptide could stimulate E7-specific through peptide loading on dendritic cells [8]. This creates a potential confound of false positive activation from unbound 9RE7 in SOMV-9RE7 synthesis that wasn’t removed during the filtration procedure. To eliminate this artifact, the 9RE7 control group was dialyzed with a 50kD centrifugal filter to examine peptide removal efficiency. From the low T cell activation treated with filtrated 9RE7 (Fig. 1G), it can be confirmed that the increased IFNγ expression in SOMV-9RE7 treated group was not an artifact from unremoved free peptide.

Delivering SOMV-9RE7 subcutaneously in TC-1 tumor-bearing mice led to significant anti-tumor effects after the first dose. Development of E7 antigen-specific T cells was confirmed in PBMC after two dose treatments, further confirming the effectiveness of the 9RE7 antigen coating strategy. SOMV-9RE7’s therapeutic effect is potentially due to direct activation of APCs in the tumor-draining lymph-node for immediate onset of adaptive immunity [27]. Due to the native inflammatory agents present on SOMV, it induced mild inflammatory responses including symptoms of redness at the injection site and weight loss of around 5%. These symptoms cleared quickly within one or two days after injection. They became much less pronounced following the second dose, meaning the mice have begun to tolerate the treatment. In future experiments, a reduced overall dose or a gradual dose escalation of SOMV can be implemented to prevent septic shock and overactive immune response.

Analyzing the immunologic profile in the TME, we observed a significant infiltration and activation of E7-specific CTLs in SOMV-9RE7 treated mice. The same trends in T cell proliferation were reflected in splenocytes. On the other hand, control treatments 9RE7 and SOMV yielded no significant amounts of E7-specific TILs (Fig. 3H) or splenocytes (Fig. 3F). Therefore, we can confidently conclude that 9RE7 antigen-display contributed to the differential E7+ T cell expansion difference between SOMV and SOMV-9RE7, which led to tumor-specific control. Interestingly, 9RE7 treated tumors showed a slight increase in CD8+ TILs (Fig. 3F), while splenocytes of SOMV treatment display an increase in CD8+ subpopulation (Fig. 4D) despite overall CD45+ CD3+ splenocyte decreased (Fig. 4B). These discrepancies in the observed local and systemic immunological profiles, however, cannot convey a comprehensive understanding of tumor-associated immune responses that correlate to tumor control. This further highlights the consistent cytotoxic lymphocyte profiles in SOMV-9RE7 immunotherapy that drive tumor-associated immunity.

Thus, we have demonstrated the successful synthesis of an MHC class I tumor-associated epitope displaying OMV vaccine with the polyarginine CPP method. Furthermore, SOMV-9RE7 exhibited immunogenic properties that activate E7-specific T cells via APC cross-presentation in vitro. Finally, administration of SOMV-9RE7 showed superior anti-tumor effects on TC-1 tumors by increasing E7-specific T cell infiltration and boosting systemic adaptive immunity. Future investigation will focus on the differences in therapeutic efficacy of 9RE7-coated *Salmonella* and SOMV to determine the more efficacious and safer platform for bacterial immunotherapy in HPV-associated and other cancer models. Previous bacteria-based combination therapy results showed highly synergistic benefits of pairing cytokine or immune checkpoint inhibitors to enhance the efficacy of bacteria therapy [8, 9, 28]. Our future experiments will dive into augmenting the CD8+ T cell-mediated tumor-specific immune cascade to determine synergistic combination therapy targets. In a phase II clincal trial of treating metastatic melanoma with modified HLA-A2*0201 bound gp100:209–217(210 M) peptide vaccine in combination with cytokine interlekin-2 (IL-2), the glycoprotein carrier can be replaced with our SOMV platform to introduce bacterial immunotherapy as a combination treatment of melanoma [29]. Due to the simplicity and efficiency of polyarginine coating strategy, it can be broadly applied to personalized neoantigen targeted therapy that utilizes next-generation sequencing to identify highly immunogenic tumor-specific neoantigen [30]. Our peptide-loaded SOMV can be seamlessly and effectively implemented in other neoantigen vaccines, such as the iNeo-Vac-P01 for pancreatic cancer[31], and the HER2-derived MHC I peptide E75 vaccine used in clinical trial for ductal carcinoma in situ [32]. Finally, small exogenous proteins and antibodies can also be displayed on OMVs using polyarginine CPPs as anchors. Engineered HPV L2 minor capsid targeting monoclonal antibodies can potentially be delivered through OMV surface presentation, which may lead to more efficient virus neutralization and offer new solutions to antibody penetration and targeting challenges [33, 34].

By implementing our innovative 9RE7 antigen coating strategy to OMV and introducing the pioneering SOMV-9RE7, we have provided a feasible and economical approach for developing bacteria-based antigen-displaying vaccines. This strategy employs a straightforward production technique, eliminating the need for designing recombinant bacterial constructs, which allows the control over the peptide to OMV ratio. Furthermore, recent studies involving OMV vaccines in phase II clinical trials have demonstrated promising results [35], indicating the translational potential of SOMV vaccines. While our SOMV-9RE7 work represents a groundbreaking advancement in bacteria-immunotherapy and vaccines for HPV-associated cancer, future preclinical and clinical research endeavors will continue to expand upon this strategy.

## Conclusions

This study is innovative in introducing the novel SOMV-9RE7 vaccine as a bacteria immunotherapy tailored to target HPV-associated cancers. We demonstrated that the combination of SOMV with 9RE7 enhances antitumor effects as well as effector T cell proliferation and activity in the tumor microenvironment and in the spleen. Our findings strongly suggest that SOMV-9RE7 vaccines represents a promising, cost-effective, and viable strategy. More clinical evidence will be needed to confirm these findings and provide a more comprehensive potential of this approach.

### Supplementary Information

**Additional file 1.** TC-1 tumor-bearing mice weight under SOMV-9RE7 treatment. Body mass of mice from each treatment group is measured twice a week. Vaccinations are given on days 7 and 14.

## Data Availability

All data relevant to the study are included in the article or uploaded as Additional information. Data and materials are available on reasonable request.

## Abbreviations

OMV: Outer membrane vesicles
LPS: Lipopolysaccharides
HPV: Human Papillomavirus
9RE7: Nine arginine residues linked to HPV E7 (aa49-57) short peptide
CPP: Cell penetrating peptide
SOMV: *Salmonella*-Derived outer membrane vesicles
TME: Tumor microenvironment
APC: Antigen presenting cells
MHC: Major Histocompatibility Complex
CTL: Cytotoxic T lymphocytes
TIL: Tumor infiltrating-lymphocytes
