# Supplementary figures and images for: Arginine-linked HPV-associated E7 displaying bacteria-derived outer membrane vesicles as a potent antigen-specific cancer vaccine
Source: J Transl Med. 2024 Apr 22;22:378. doi: 10.1186/s12967-024-05195-7 (PMC11036690; doi:10.1186/s12967-024-05195-7)

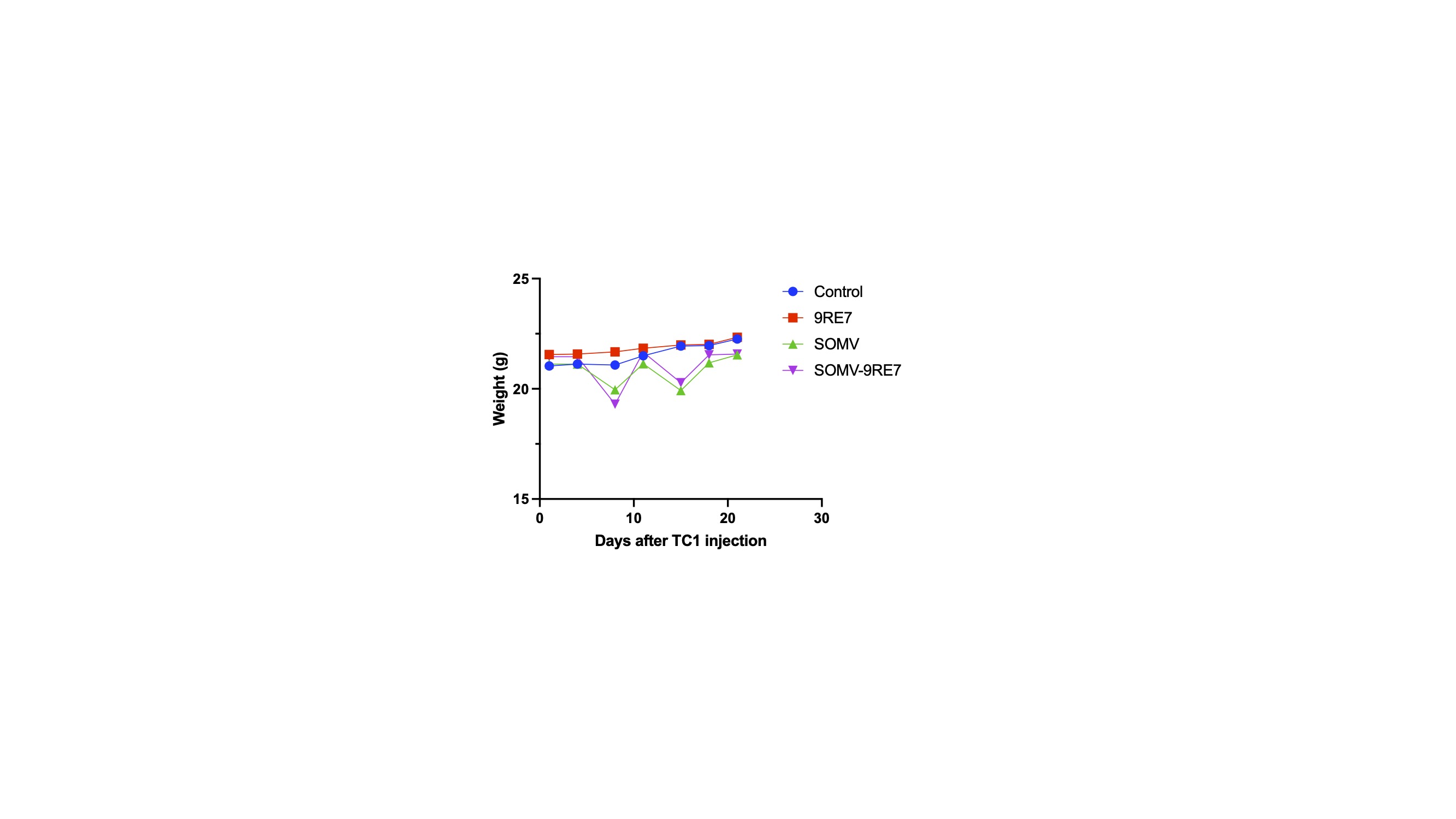

Supplement: Supplementary file 1 — Additional file 1. TC-1 tumor-bearing mice weight under SOMV-9RE7 treatment. Body mass of mice from each treatment group is measured twice a week. Vaccinations are given on days 7 and 14. [file 12967_2024_5195_MOESM1_ESM.jpg]
